# Evaluation of Live Attenuated H7N3 and H7N7 Vaccine Viruses for Their Receptor Binding Preferences, Immunogenicity in Ferrets and Cross Reactivity to the Novel H7N9 Virus

**DOI:** 10.1371/journal.pone.0076884

**Published:** 2013-10-09

**Authors:** Qi Xu, Zhongying Chen, Xing Cheng, Lucy Xu, Hong Jin

**Affiliations:** MedImmune LLC, Mountain View, California, United States of America; University of Massachusetts Medical Center, United States of America

## Abstract

Live attenuated influenza vaccine (LAIV) candidates of the H7 subtype, A/Netherlands/219/03 (H7N7, NL03 *ca*) and A/chicken/British Columbia/CN-6/2004 (H7N3, BC04 *ca*), were evaluated for their receptor binding specificity and immunogenicity in ferrets. The BC04 *ca* virus exhibited α2,3-SA and α2,6-SA dual receptor binding preference while the NL03 *ca* virus preferentially bound to α2,3-SA. Substitution of the Q226 and G228 (Q-G) by the L226 and S228 (L-S) residues in the HA improved binding to α2,6-SA for NL03 *ca*. The vaccine viruses with L-S retained the attenuation phenotype. NL03 L-S *ca* replicated more efficiently than the original NL03 *ca* virus in the upper respiratory tract of ferrets, and induced higher levels of humoral and cellular immune responses. Prior vaccination with seasonal LAIV reduced H7-specific antibody responses, but did not reduce the H7N7 vaccine mediated protection against a heterologous H7N3 BC04 *wt* virus infection in ferrets. In addition, the H7N3 and H7N7 vaccine immunized ferret sera cross reacted with the newly emerged H7N9 virus. These data, in combination with the safety data from previously conducted Phase 1 studies, suggest that these vaccines may have a role in responding to the threat posed by the H7N9 virus.

## Introduction

Influenza A viruses cause yearly seasonal influenza epidemics and occasional influenza pandemics when a novel influenza virus with an antigenically shifted hemagglutinin (HA) emerges in humans resulting in widespread infection, high morbidity and high mortality [1]. Avian influenza H7 subtype viruses have caused occasional human infections and have thus raised pandemic concerns. In the past, most of the reported H7 cases had a history of direct physical contact with H7-infected poultry or seals. In the spring of 2003, a large outbreak of H7N7 infection in humans occurred in the Netherlands [2,3]. The highly pathogenic avian influenza (HPAI) H7N7 virus with multibasic amino acids at the HA cleavage site infected more than 30 million birds in poultry farms. Among the estimated 4,500 people who were exposed to infected poultry, 89 people had laboratory-confirmed H7N7 virus infection with one fatality. Most infected individuals exhibited conjunctivitis or respiratory symptoms with limited human-to-human transmission. In the spring of 2004, an H7N3 outbreak occurred in British Columbia, Canada [4,5]. A total of 57 workers in a poultry farm were reported to have conjunctivitis or influenza-like symptoms. Among the viruses isolated from the outbreak, A/chicken/British Columbia/CN-7/2004 was also identified as an HPAI virus due to the presence of the multibasic amino acids at the HA cleavage site, while A/chicken/British Columbia/CN-6/2004 was confirmed to be a low pathogenic avian influenza (LPAI) virus due to the monobasic amino acid at the HA cleavage site [6]. Human infections of H7N2 subtype in North America were also reported [7]. In the spring of 2013, H7N9 infections in humans were first reported in China. The discovery of human infections caused by the viruses in China is a major public health concern as it is unlikely that there will be pre-existing immunity to this subtype in the population [8]. The H7N9 virus is low pathogenic in the avian host, but is highly pathogenic in humans. As of August 2013, there were 132 reported human infections including 43 fatal cases since February 2013.

Vaccination is the most effective method to prevent influenza infection. Live attenuated influenza vaccines (LAIV) [9] have the ability to provide protection against antigenically drifted strains [10,11,12]. Previously, in preparation for a potential H7 pandemic, both H7N7 (A/Netherlands/219/03, NL03 *ca*) and H7N3 (A/chicken/British Columbia/CN-6/2004, BC04 *ca*) LAIVs were generated and evaluated in pre-clinical studies and phase I clinical trials; the H7N3 vaccine candidate was found to be more immunogenic than the H7N7 vaccine [6,13,14,15].

As influenza viruses that have caused past pandemics had receptor binding preference for α2,6-linked sialic acid (α2,6-SA), it is generally believed that avian viruses need to acquire binding specificity to these human cell receptors to establish efficient human-to-human transmission. Most avian viruses, however, preferentially bind to α2,3-SA receptors. Candidate vaccine viruses that contain the HA and NA protein from an avian influenza virus thus may not replicate efficiently in the mammalian upper respiratory tract and may be less immunogenic as a result. Recently, we demonstrated that the H2N2 (A/Ann Arbor/6/60) and H6N1 (A/Teal/Hong Kong/97) LAIV viruses with dual receptor binding to both α2,3-SA and α2,6-SA, as specified by L226 and S228 residues in the HA gene, grew efficiently in eggs and induced higher antibody levels in ferrets than those with Q226 and G228 residues that resulted in preferential binding to α2,3-SA [16]. The H7 vaccine viruses we generated previously contain the original Q226 and G228 in the HA. In this study, we made H7 vaccine virus variants by introducing Q226L and G228S substitutions in the HA and investigated their impact on receptor binding preference, vaccine virus replication, antigenicity, immunogenicity and protection against a wild-type H7N3 virus challenge infection in ferrets. In addition, the influence of seasonal LAIV vaccination on the immunogenicity and protective activity of the H7N7 pandemic vaccine variant was also evaluated. The extent to which the H7N3 and H7N7 immunized ferret sera cross reacted with the newly emerged H7N9 virus was also determined in order to evaluate whether these vaccine viruses might have the potential to provide protective immunity against this strain.

## Materials and Methods

### Generation of reassortant vaccine viruses

Influenza H7N7 A/Netherlands/219/03 (NL03 Q-G) *ca* with the multibasic cleavage site removed and H7N3 A/chicken/BC/CN-6/04 (BC04 Q-G) *ca* vaccine viruses were generated previously [13,14]. H7N9 A/Anhui/1/2013 (H7N9) reassortant vaccine virus (AH13 *ca*) was also generated by reverse genetics using the HA (accession# 439507) and NA (accession# 439509) sequences from the EpiFlu database of the Global Initiative on Sharing All Influenza Data (GISAID). Wild type A/chicken/BC/CN-6/04 is a low pathogenic virus and is handled at the BLS2 facility. The HA mutations were introduced into the live attenuated H7N7 or H7N3 vaccine viruses by reverse genetics and sequences were verified. All viruses were propagated in the allantoic cavity of 10- to 11-day-old embryonated chicken eggs (Charles River SPAFAS). Virus titers were determined in Madin-Darby Canine Kidney (MDCK) cells and expressed as fluorescent focus units (FFU) per ml.

### Receptor binding assay

Receptor binding preference of the vaccine viruses was examined by the glycan binding assay [17]. Two-fold serially diluted virus in PBS with 1% (w/v) BSA (PBS-BSA) in duplicates were added to glycan-bound wells at concentration of 2.4 µM and incubated overnight at 4°C. The plates were incubated with 1:4000 diluted sheep NL03 H7 HA-specific antiserum (MedImmune) overnight at 4°C followed by incubation with 1:2000 dilution of rabbit anti-sheep IgG-HRP (Dako, *Carpenteria*, CA). The reactions were detected by TMB (Thermo, Fisher Scientific) and the absorbance at 450 nm was determined using a SpectraMax plate reader (Molecular Devices). Receptor binding preference of recombinant viruses was also confirmed by hemagglutination of sialic acid-specific red blood cells (RBC) by the method described previously [18,19].

### Ferret studies

All animal study protocols were approved by MedImmune’s Institutional Animal Care and Use Committee (IACUC) and performed in an AAALAC certified facility under protocol number ACF-081-007, 017 and 029. Eight to ten weeks-old male and female ferrets (n = 3-5/group) from Simonsen Laboratories (Gilroy, CA) were used in the studies. To examine the attenuation phenotype, ferrets were inoculated intranasally with 7.0 log_10_FFU of virus per 0.2 ml dose. At 3 days post inoculation, lungs and nasal turbinates (NT) were harvested, viral titers in the respiratory tissues were determined in eggs and expressed as 50% egg infectious dose (EID_50_).

For immunogenicity and protection from wild-type challenge studies, ferrets were inoculated with 7.0 log_10_FFU as described above and blood samples were obtained on days 8 and 14 post-vaccination. Hemagglutination inhibition (HI) and microneutralization (MN) assays were performed [20,21]. The T cell response in peripheral blood mononuclear cells (PBMC) was evaluated by an enzyme-linked immunospot (ELISPOT) assay [22,23] using recombinant H7 HA of H7N7 NL03 obtained from Protein Sciences Corporation or H7N7 NL03 (Q-G) *ca* virus as antigen.

The effect of seasonal vaccination on H7N7 vaccine induced immune responses and protection was also examined. Ferrets were first intranasally inoculated with PBS or LAIV containing A/New Caledonia/20/1999 (H1N1) *ca*, A/Wisconsin/67/2005 (H3N2) *ca* and B/Malaysia/2506/2004 ca at 7.0 log_10_FFU per strain in 0.2 ml. Four weeks later, they were inoculated with 0.2 ml of 7.0 log_10_FFU of NL03 L-S or PBS. The blood samples were obtained on days 8 and day 14 after the second dose, and serum antibody and T cell responses in PBMC were determined. On day 28 post-dose 2, the ferrets were challenged with wild type (wt) A/chicken/BC/CN-6/04 (BC04 *wt*) at a dose of 7.0 log_10_FFU per 0.5 ml and sacrificed three days post challenge to determine viral titers in the NT and lungs.

## Results

### Receptor binding specificity of H7 vaccine viruses

**Figure 1:**
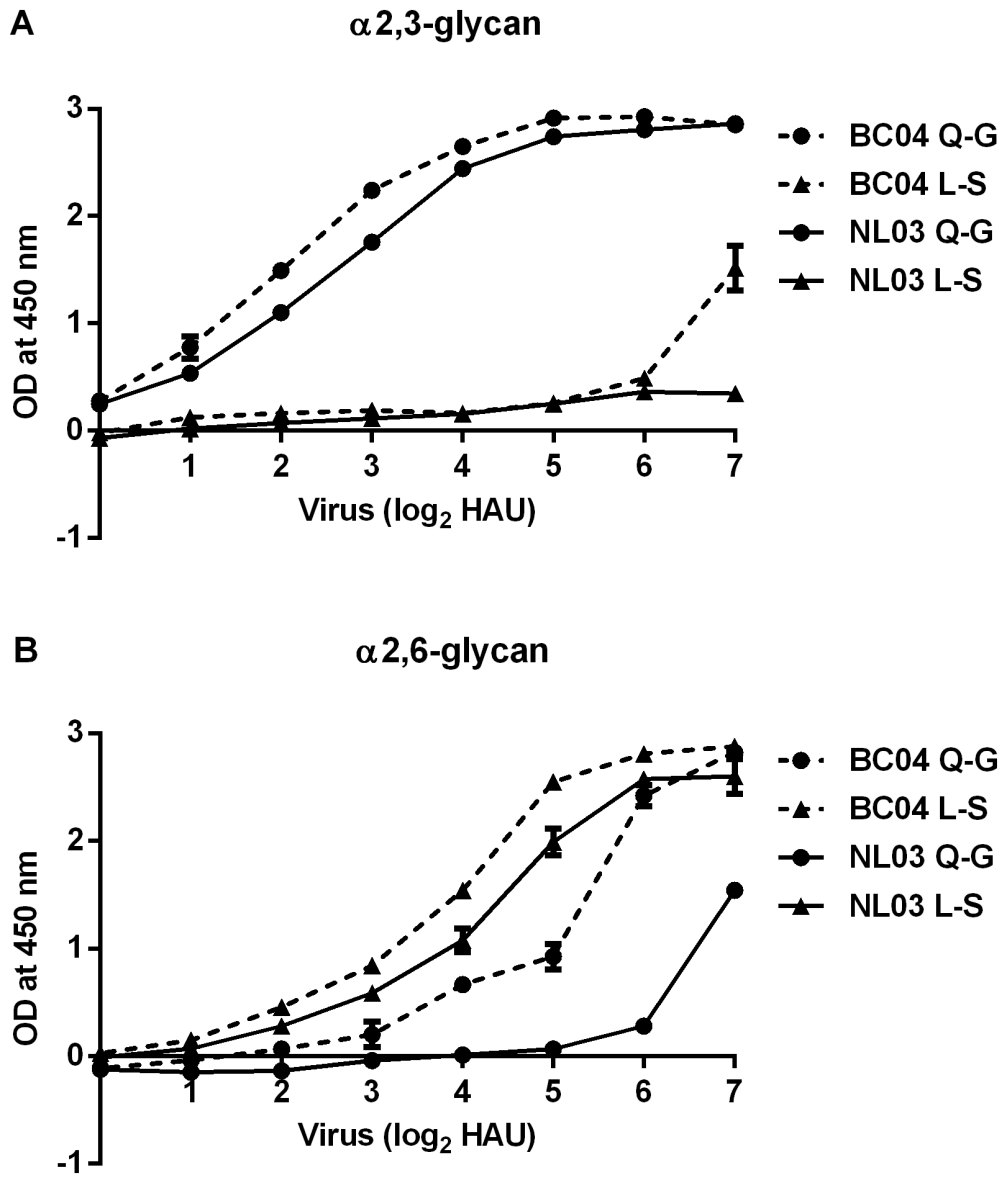
Glycan binding specificity of the H7 vaccine viruses to biotinylated 3'S-Di-LN-LC-LC (A) or 6'S-Di-LN-LC-LC (B). The binding ability of the 2-fold serial diluted virus in HA unit to immobilized glycan was detected by ELISA assay using NL03 HA specific sheep antiserum. The data are presented as geometric mean of optical density (OD) at 450 nm ± standard error (SE) of two independent samples.

The H7N7 NL03 *ca* and H7N3 BC04 *ca* vaccine viruses with HA Q226L and G228S substitution were named as NL03 L-S or BC04 L-S. The viruses with the L-S change grew equally well in eggs, reaching titer of ~8.5 log_10_FFU/ml. By glycan binding assay, the original BC04 and NL03 *ca* vaccine viruses with Q226 and G228 (Q-G) bound to α2,3-glycan (Figure 1A). BC04 Q-G bound to α2,6-glycan at a greater level than NL03 Q-G (Figure 1B). Both NL03 L-S and BC04 L-S preferentially bound to α2,6-glycan.

The glycan-binding data was verified using an alternative method of hemagglutination of α2,3-SA or α2,6-SA resialylated chicken red blood cells (RBC) (Table 1). BC04 Q-G bound to both types of sialic acids but with a higher binding capacity to α2,3-SA;BC04 L-S had similar binding preferences for both types of receptor. NL03 Q-G only bound to α2,3-SA, while NL03 L-S gained binding capacity to α2,6 SA. These data confirmed that both H7 influenza viruses gained receptor binding to α2,6-SA through the L-S changes in the HA [16].

**Table 1:** Receptor binding specificity and replication of H7 vaccine viruses in the respiratory tract of ferrets.

strain	H7 HA (H3#)	Receptor binding specificity^1^			Virus titer^2^	
	226-228	*cRBC*	*α2,3*	*α2,6*	NT	Lung
BC04	Q-G	128	64	8	5.8±0.1	<1.5
	L-S	128	64	64	5.9±0.2	<1.5
NL03	Q-G	128	64	<2	1.8±0.2	<1.5
	L-S	256	128	64	5.0±0.1*	<1.5

^1^ Chicken RBCs were completely desialylated and resialylated with indicated glycans. Virus binding specificity was determined by hemagglutinating regular chicken RBC (cRBC) or receptor specific RBC. The detection limit is HA titer 2.

^2^ Groups of three ferrets were intranasally inoculated with the indicated H7 vaccine variants. Three days post inoculation, viral titers in NT and lungs were measured by the EID50 assay and the titer is expressed as GMT± SE log_10_EID_50_/g. The detection limit is 1.5 log_10_ EID_50_/g * indicates statistically significant (P<0.05) difference in virus titer compared to the Q-G variant of the same strain by *t* test.

### The substitutions of residues 226 and 228 did not affect vaccine virus attenuation phenotype

The attenuation phenotype of the *ca* vaccine viruses is defined by preferential replication in the upper respiratory tract compared to the lower respiratory tract of ferrets. As shown in Table 1, both the H7N3 BC04 and H7N7 NL03 L-S variants did not replicate in the lungs of infected ferrets, confirming that changing the receptor specificity did not affect the attenuation phenotype of the vaccine viruses. BC04 Q-G and L-S *ca* viruses replicated efficiently in the NT at levels of >5 log_10_EID_50_/g tissue. NL03 Q-G replicated poorly in the NT (1.8 log_10_ EID_50_/g) but the NL03 L-S replicated well in the NT reaching a titer of 5.0 log_10_EID_50_/g. Thus, acquisition of virus receptor binding to α2,6-SA improved NL03 *ca* virus replication in the upper respiratory tract of ferrets.

### Immunogenicity and antigenicity of the H7 vaccine viruses

The immunogenicity of BC04 *ca*, NL03 *ca* and NL03 L-S were compared by measuring serum HI antibody titers from immunized ferrets after a single dose of vaccine (Table 2). BC04 *ca* was more immunogenic than NL03 *ca*, eliciting an HI geometric mean antibody titer of 256. NL03 *ca* was less immunogenic than NL03 L-S, its HI antibody titer (20) was 4-fold lower than the NL03 L-S titer (81). NL03 L-S immunized ferret sera also cross reacted with the heterologous BC04 *ca* although the titers were again ~3-4-fold lower. Microneutralization (MN) assay against different NL03 *ca* viruses was also performed (Table 2). BC04 *ca* immunized ferret serum could neutralize heterologous viruses at about 3-4 fold lower levels. BC04 L-S elicited antibody responses at a level similar to BC04 *ca* (data not shown). Consistent with the HI data, the NL03 L-S variant induced a higher level of neutralizing antibody against the homologous virus (GMT 202) than the NL03 Q-G (GMT 63), and the antisera cross reacted well with the heterologous BC04 *ca* virus (GMT 101). These results indicate that vaccine viruses with better replication efficiency in the upper airway induced higher serum antibody responses in ferrets and that the 226 and 228 residues at the receptor binding site did not significantly alter viral antigenicity.

**Table 2:** Serum antibody titers of the ferrets immunized with H7 vaccine viruses.

Vaccine strain	H7 HA (H3#) 226-228	HI Ab GMT against the indicated virus			Nt Ab GMT against the indicated virus		
		BC04	NL03	NL03 L-S	BC04	NL03	NL03 L-S
BC04	Q-G	**256**	8	25	**320**	40	101
NL03	Q-G	20	**20**	81	80	**63**	202
	L-S	40	20	**81**	101	80	**320**
AH13	L-G	51	6	25	101	40	101

Ferrets were immunized with the indicated H7 vaccine viruses. Serum antibody titers at day 14 post vaccination were determined by the HI and microneutralization assays. The titers are presented as geometric mean titer (GMT). Homologous antibody titers are bolded and underlined.

H7N3 BC04 and H7N7 NL03 immunized ferret sera were further evaluated for their cross reactivity against the newly emerged H7N9 A/Anhui/1/2013 strain (AH13). The BC04 Q-G HI and neutralizing Ab GMT against the AH13 strain (51 and 101) was approximately 3 to 4-fold lower than the titer seen for the homologous strain (256 and 320). The NL03 Q-G and NL03 L-S immunized ferret sera cross reacted to H7N9 AH13 at a level of approximately 3-fold lower than what was seen for the homologous strain by both HI and microneutralization assays (Table 2).

**Figure 2:**
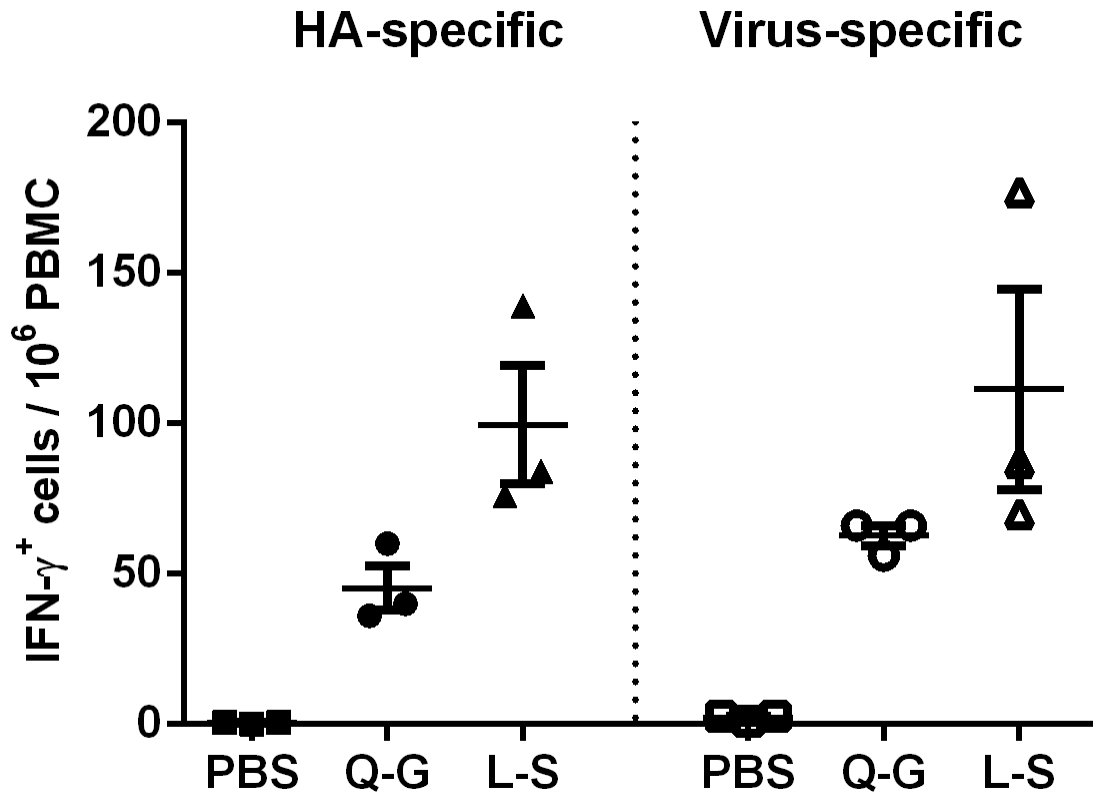
NL03 specific IFN-γ secreting T cell responses in ferret PBMC on day 8 post vaccination stimulated with either NL03 HA or virus. The solid lines represent geometric mean ± standard error for the group.

Evaluation of vaccine mediated cellular immune responses focused on the comparison of NL03 Q-G and L-S as BC04 L-S did not have a significant impact on viral receptor binding preference (Figure 2) or immunogenicity (data not shown). NL03 L-S elicited a higher level of H7 HA specific T cell response than NL03 Q-G (P=0.062). The number of IFN-γ secreting cells in the blood stimulated by the intact virus in the assay was only slightly higher, likely due to the detection of the responses directed against the NA surface protein in the virions, the other internal proteins such as the NP and M1 protein mediated T cell responses may not be detected as the stimulating viruses were not disrupted. Virus specific IFN-γ secreting cells were higher for NL03 L-S immunized ferrets than for the NL03 Q-G immunized animals, but the values were not statistically different because of the small group size.

### Seasonal LAIV vaccination does not affect H7N7 *ca* vaccine mediated protection

**Figure 3:**
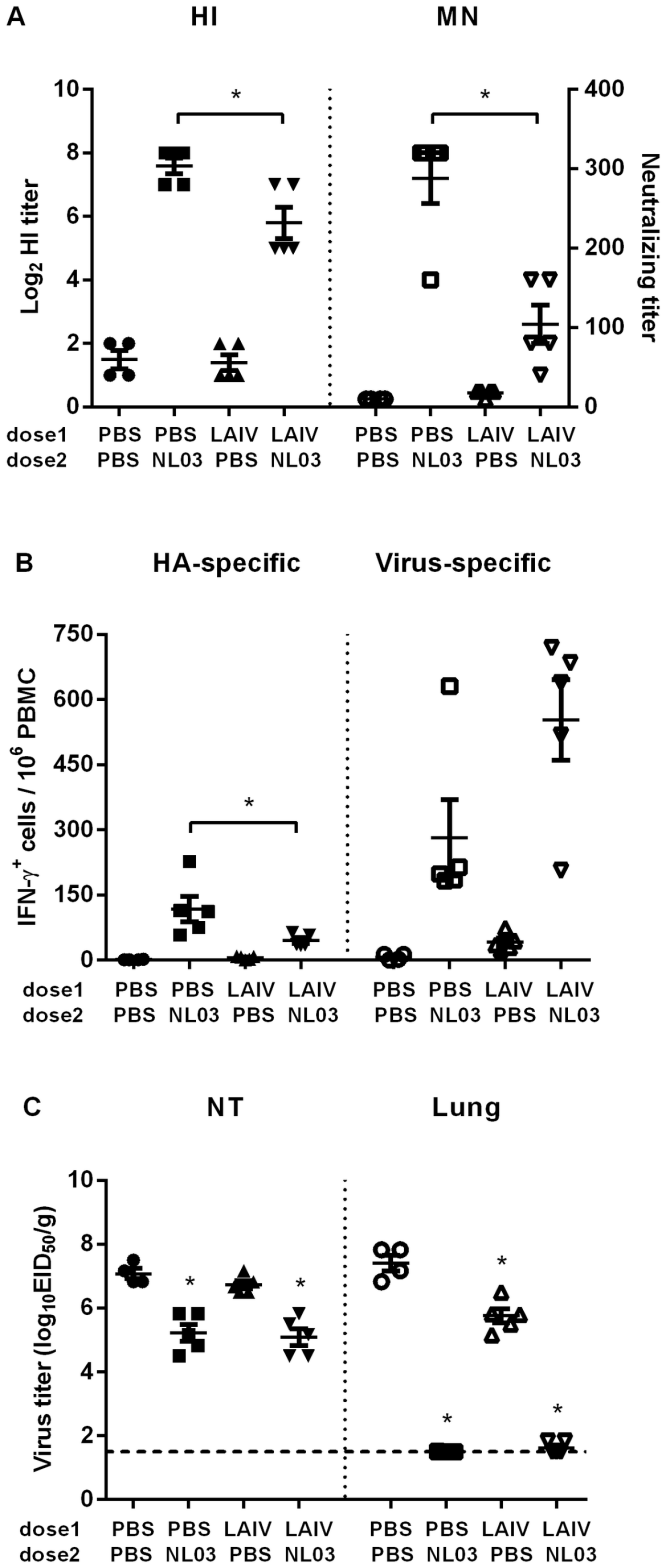
Seasonal LAIV on NL03 L-S vaccine virus induced immune responses and protection against a heterologous virus challenge infection. **A**. Ferrets were immunized with the indicated viruses and serum antibody titers on day 14 post the second vaccine were determined by the HI assay or MN assay with NL03 L-S vaccine virus. The data are expressed as log_2_ HI or neutralizing antibody titers, respectively. **B**. HA- or virus-specific IFN-γ secreting cells in PBMC on day 8 post vaccination. The solid lines represent geometric mean ± SE for the group. **C**. The ferrets were challenged with BC04 wt 28 days post the second dose. Challenge virus titer in NT and lung tissues at 3 days post challenge was expressed as log _10_EID_50_/g (GMT ± SE). The dashed line indicates limit of detection. * indicates statistically significant (P<0.05) difference by *t* test.

The influence of pre-existing immunity to seasonal influenza viruses on the immunogenicity of the live attenuated H7N7 vaccine was evaluated (Figure 3). Ferrets were first immunized with seasonal LAIV or PBS, followed by intranasal administration of either PBS or NL03 L-S a month later. Serum HAI antibody elicited by seasonal LAIV did not cross react with the H7N7 NL03 *ca* virus. The animals that previously received seasonal LAIV had reduced antibody response to the H7N7 vaccine compared to the naïve ferrets, exhibiting approximately 3-fold reductions in HI and MN antibody titers (Figure 3A). The H7-specific IFN- γ secreting T cells examined at day 8 post H7N7 NL03 vaccination were detected in the naïve animals but not in the LAIV vaccinated animals (Figure 3B). However, the number of virus-specific IFN-γ secreting cells was higher in the LAIV primed animals compared to the animals that received NL03 *ca* only, reflecting recall responses to the other viral antigens.

The H7N7 NL03 L-S immunized ferrets, primed with or without LAIV, were examined for protection against heterologous BC04 *wt* virus challenge infection (Figure 3C). A heterologous BC04 *wt* virus (BSL2 agent) was used as the challenge virus as studies conducted with the highly pathogenic NL03 *wt* virus required BSL3 facilities not available at MedImmune. The BC04 *wt* virus replicated to a titer of approximately 7.0 log_10_EID_50_/g in both the NT and lung tissues of the PBS immunized ferrets. Seasonal LAIV did not inhibit challenge virus replication in NT, but reduced challenge virus titers in the lung by approximately 50-fold. The animals that received LAIV followed by the H7N7 NL03 vaccine were protected against BC04 *wt* replication in the respiratory tract at similar levels compared to the animals immunized with NL03 *ca* only. The challenge virus titers in the NT were reduced by approximately 100-fold and no virus were detected in the lungs of the ferrets that were vaccinated with a single dose of NL03 L-S regardless of their LAIV priming status. These data indicated that previous seasonal LAIV immunization did not diminish H7N7 vaccine induced protection in ferrets.

## Discussion

The HA-mediated viral entry through cell surface sialyated glycans is important for viral replication, virulence and transmission of influenza viruses [24]. Live attenuated influenza vaccines that can bind to both α2,3-SA and α2,6-SA are more likely to grow to the high titers in embryonated chicken eggs required for commercial manufacturing and to replicate efficiently in the human respiratory tract resulting in induction of protective immune responses. The H7N7 NL03 virus predominantly bound to α2,3-SA and replicated at a lower level in the respiratory tract of ferrets; this may explain why it appeared to be less immunogenic than the H7N3 BC04 *ca* vaccine in preclinical and clinical studies [13,14,15]. Here, we reported that the L-S change in the HA of NL03 enabled it to bind to both α2,3-SA and α2,6-SA, which improved viral replication in the upper respiratory tract (NT) of ferrets and resulted in increased immunogenicity compared to the original NL03 *ca* vaccine.

The HA residues 226 and 228 are located in the 220 loop of the HA receptor binding site [24] and their effect on receptor binding preference has been studied extensively for various subtypes of influenza viruses. The early H2 and H3 human viruses established the α2,6-SA binding to the human host by acquiring the L226-S228 residues [25,26,27]. The L226 residue was also found to specify HA binding to α2,6-SA for H4, H5 and H9 viruses [28,29,30,31,32]. We previously showed that introduction of the L226 and S228 to the HA proteins of H2 and H6 vaccine viruses derived from avian viruses, or to viruses adapted for growth in eggs, promoted virus binding to both α2,3-SA and α2,6-SA and improved vaccine virus immunogenicity in ferrets [16]. Although both the BC04 and NL03 viruses have the same Q226 and G228, the BC04 virus with these amino acids can bind to both α2,3-SA and α2,6-SA. The L-S change, therefore, had minimal impact on receptor binding for the BC04 *ca* virus. The North America lineage H7N3 BC04 strain with Q-G, also replicated well in the respiratory tract of ferrets and humans [15]. The H7N2 and H7N3 viruses of North America lineage have been shown to have increased binding capacity to α2,6-SA [33]. Binding of H7N2 strains (such as A/NY/107/2003) to α2,6-SA is mediated by the loss of 8 amino acids in the 220 loop [34]. Thus, in addition to the 226 and 228 residues, other amino acids in the HA protein also influence viral receptor binding. The L-S change in the HA of NL03 significantly improved its binding capacity to α2,6-SA. The dual binding preference of NL03 L-S was better demonstrated by the RBC binding assay than the glycan assay because of structural differences in the sialic acid molecules used in these two assays and due to differences in assay sensitivity. The newly emerging H7N9 viruses predominantly contain the L226 residue that mediates binding to α2,6-SA [16]; this may be contributing to the numerous cases of transmission to humans that have been documented to date.

Consistent with the replication of vaccine viruses in ferret NT, the NL03 L-S variant induced higher antigen-specific antibody and T cell responses than the original NL03 *ca*. Previous infection with one subtype of influenza viruses may induce heterosubtypic immunity against infection with an antigenically unrelated subtype [35]. In this study, we showed that seasonal LAIV vaccination slightly reduced H7N3 BC04 *wt* virus titer in the lungs (Figure 3C) due to this heterosubtypic immunity. Although seasonal LAIV vaccination reduced H7N7 specific immune responses because of the inhibition of H7 *ca* virus replication in the upper airway, all the H7N7 NL03 L-S *ca* vaccinated ferrets were completely protected from the replication of the heterologous H7N3 BC04 *wt* challenge virus challenge in the lung. The H7N3 BC04 and H7N7 NL03 vaccines are antigenically distinct, exhibiting approximately 3-fold difference in serum antibody cross reactivity. Similarly, the newly emerged H7N9 AH13 virus and the H7N7 NL03 L-S or H7N3 BC04 had approximately 3-fold difference in serum antibody cross reactivity. Based on the cross protection data obtained from the H7N7 NL03 L-S against BC04 wt virus challenge, it is predicted that the existing H7N3 or H7N7 *ca* vaccines would provide some degree of protection against H7N9 virus. As master virus seeds for these vaccines have already been produced and the safety profile demonstrated in the previous small phase 1 studies, manufacturing with the exiting vaccine virus will save at least 3 months in vaccine product delivery.

We recently conducted the ferret immunogenicity study for the H7N9 AH13 *ca* virus. The H7N9 AH13 vaccine virus elicited neutralizing antibody titer of 320 against the homologous virus. Due to the lack of the BSL3 facility, we were not able to conduct the wt H7N9 AH13 virus challenge study for this report. Should the H7N9 virus become a pandemic threat, the H7N9 *ca* virus can now be manufactured to respond to the pandemic.
